# Soft actor critic-based performance optimization for IRS-aided cognitive radio systems

**DOI:** 10.1038/s41598-026-49465-4

**Published:** 2026-05-05

**Authors:** Rna Ghallab, Ahmed Abdrabo, Ibrahim Elashry

**Affiliations:** Electrical and Electronic Engineering Department, College of Engineering, Kafrelsheikh University, Kafr El-shaikh, 33516 Egypt

**Keywords:** Intelligent reflective surfaces, Cognitive radio, Deep reinforcement learning, Engineering, Mathematics and computing

## Abstract

An intelligent reflective surface (IRS)-assisted cognitive radio (CR) multiple-input multiple-output (MIMO) communication system is considered. Incorporating cognitive radio and IRS capabilities into such a system yields significant improvements in system performance, including energy efficiency (EE) and receiver quality of service (QoS). For enhancing the attainable rate of secondary users (SU) without exceeding the interference temperature limit (IT) on the primary users (PU), a non-convex optimization problem is formulated, which is usually solved by means of alternative optimization (AO) methods such as block coordinate descent (BCD) algorithms. In this paper, we focus on deep reinforcement learning (DRL) approaches, specifically, the soft actor-critic (SAC) algorithm, to solve this optimization problem. For comparison, all simulation figures will be composed of a BCD benchmark beside the SAC curves. In addition, a 16-element MIMO antenna array for the secondary transmitter (ST) base station is proposed, designed, fabricated, and tested, yielding a 90% radiation efficiency with perfect impedance matching and acceptable return losses.

## Introduction

The massive growth in the number of mobile users and wireless devices connecting to the network in beyond-5G wireless communication systems boosts the demand for enhanced spectral efficiency, low latency, high data rates, improved QoS, and higher energy efficiency^1^. Such a rapid increase needs tremendous spectrum resources, and due to the stationary way in which the radio spectrum is organized, improving the utilization of spectrum resources is required. It is known that spectrum is scarce, expensive, and it is necessary to devise flexible and dynamic spectrum access techniques that enable optimal usage of such a valuable resource^2^.

One of the most important ways to address this spectrum scarcity issue is to utilize higher frequency bands, such as millimeter-wave bands. The sub-6 GHz band is already congested and cannot provide any additional bandwidth, while the mm-Wave bands provide greater bandwidth and have sufficiently small wavelengths, which are more suitable for the massive MIMO technology. A contemporary key solution to the spectrum scarcity problem is to exploit the cognitive radio (CR) technique^3^. An underlay spectrum sharing CR is an efficient way to raise the spectral efficiency of a wireless communication system by making the secondary users (SU) capable of spectrum sharing with the primary users (PU) while controlling the interference leakage under certain QoS constraints imposed by the PU^4^.

The main challenge in the CR deployment is the process of spectrum sensing (SS) in which the SU should estimate the channels accurately in order to detect a spectrum hole, which indicates that a PU of a high priority is not active. Consequently, this grant the SU the right to broadcast on these licensed parts of the spectrum when the PU is not utilizing them. The most common and conventional SS method is the energy detection (ED) technique, which is simple and easy to implement but has a poor performance at low SNR values^5^. Statistical detection methods employ statistical information of the primary signal to improve their state sensing result or occupancy determination. Also, machine learning-based techniques such as deep reinforcement learning algorithms show clear improvements in performance.

Although applying the CR technology enhances the spectrum efficiency, the performance upgrades for both the PU and the SU are mostly inconsistent, making it challenging implement and maintain the rates of both of them simultaneously^6^. This problem may be mitigated by incorporating the IRS into CR systems because the IRS can effectively assist in boosting the SU signal intensity in a desired direction and mitigating the co-channel interference that disturbs the PU^7^. IRS is a promising technology because of its low power consumption and ease of implementation. An IRS surface, composed of some reflecting elements that are made of unique materials, can efficiently improve the power consumption of a wireless communication system by regulating the reflecting coefficients and angles of the incident wave and reflecting it passively in a dedicated direction^8^. The IRS passive beamforming and the active transmit precoding (TPC) at the base station (BS) are jointly adapted to guarantee certain performance improvements, including boosting the channel capacity and the security rate of the physical layer, in addition to minimizing the transmission latency and the required transmit power^9^.

In IRS-assisted wireless systems, it is demanding to estimate the BS and user channels that are associated with the IRS due to the fact that the IRS is fully passive and not capable of sending or receiving pilot symbols, but the dysfunctional collaboration between the PU and the SU exacerbates this issue. Thus, estimating the cascaded BS-RIS-user channels is more economical^10,11^. Thus, inspired by the synergies between CR and IRS technologies, this work considers an IRS-aided mm-wave MIMO CR network adopting the perfect CSI case to upgrade the performance of the secondary network (SN) in an underlay setup, adhering to certain QoS constraints imposed by the primary network (PN). The fully-passive IRS channel estimation can be applied in such a TDD system by estimating the cascaded channel state information in one direction (downlink/uplink), and exploiting the channel reciprocity property. The BS receives the transmitted orthogonal pilot signals by the users from both the direct channel coefficients and the cascaded channel coefficients. Since the IRS reflects the incident signals based on a predefined pattern, the BS can estimate the cascaded channel coefficients and determine the optimum reflection phases, and then, it is transmitted to the IRS smart controller relying on the back-haul link.

In this paper, we concentrate on enhancing the maximum achievable rate for the secondary users. The SU rate maximization process is achieved by jointly optimizing the transmit power of the ST base station and the IRS’s reflecting-coefficients, without exceeding the IT limit on the PUs. This forms a non-convex optimization problem, which was mainly addressed by means of the alternative optimization (AO)-based techniques such as the BCD algorithm, which requires high computational complexity and complicated mathematical transformations. DRL-based algorithms, mainly the DDPG-based algorithm, that achieve a high learning efficiency with an acceptable reward variance, have been recently proposed to address this challenge. In this paper, we propose the SAC-based algorithm, which can achieve a comparable SU rate, learns faster, and gain a higher average reward with less variance if compared to the DDPG-based algorithm.

The paper’s remaining sections are constructed as follows. In Section II, a brief summary of earlier relevant work is presented. Section III illustrates the proposed IRS-aided CR system model and the resultant optimization problem. In Section IV, we discuss the perfect CSI case and how to use the SAC-based algorithm to solve the formulated optimization problem efficiently. Section V demonstrates the proposed antenna array design for the ST base station and how to be practically fabricated to achieve an acceptable radiation efficiency. We evaluate the system performance for the proposed SAC-based algorithm compared to the BCD algorithm in Section VI. Finally, Section VII concludes the paper and provides potential areas for our next research.

### *Notations*

In this paper, the real and complex number sets are symbolized by $\:\mathbb{R}$ and $\:\mathbb{C}$, respectively. Scalars are written by regular lower-case letters, vectors by bold lower-case letters, and matrices by bold upper-case letters. For vector **b**, diag(**b**) stands for a diagonal matrix having diagonal elements that match **b**. For matrix **B**, its rank, Frobenius norm, trace, transpose, and complex conjugate transpose are indicated by rank(**B**), ||**B**||, tr(**B**),$\:{\:\mathbf{B}}^{\mathrm{T}}$ and $\:{\mathbf{B}}^{\dagger}$, respectively. **B** $\:\succcurlyeq\:0$ indicates that **B** is a positive semidefinite (**PSD**) matrix, and vec(**B**) represents a column vector by stacking all the elements of **B**. While **B**$\otimes$**A** represents the Kronecker product of **B** and **A**. The distribution of a circularly symmetric complex Gaussian random variable with mean *µ* and variance $\:{\sigma\:}^{2}$ is represented by $\:\mathcal{C}\mathcal{N}$(µ, $\:{\sigma\:}^{2}$). The N-dimensional zero and identity matrices are denoted by $\:{0}_{\boldsymbol{N}}\:$and $\:{\boldsymbol{I}}_{\boldsymbol{N}}$, respectively.

## Related work

This section provides a quick overview of the relevant research, and the impact of incorporating the CR and IRS technologies into the existing MIMO communication systems is shown. Multiple CR beamforming approaches have been developed under the premise of the perfect knowledge of CSI at the SU-TX side. In^12^, only one SU is capable of utilizing the primary spectrum by limiting the interference contributions to each PU in the network. On the other hand, for enabling multiple SUs, different beamforming approaches are suggested to enhance the achievable rate for each SU in^13,14^. Nevertheless, most practical scenarios encounter the challenge of channel uncertainty where the CSI is not known. Robust stochastic beamforming approaches that can counter this challenge are proposed in^15–17^. While, the worst-case approaches are proposed and discussed in^18–20^. Recently, IRS-aided wireless communications have been widely investigated. In^21^, the attainable rate maximization problem for OFDM systems is illustrated. For a multiuser MIMO system, the IRS concept is utilized to enhance the SINR as in^22^. EE is mainly enhanced by designating the appropriate transmitting power and carefully designing the IRS-reflecting coefficients assisted by precoding^23^. Channel estimation in IRS-assisted systems is obviously a difficult challenge since the IRS usually consists of passive reflective elements, which are not capable of performing active transmission or reception. According to the binary reflection method suggested in^24^, the IRS controller activates each reflective element respectively, while maintaining the other reflective elements deactivated. Then, the BS can easily successively estimate the BS-IRS-user cascaded channels. Splitting the IRS into several sub-surfaces, and each sub-surface has a common reflection coefficient as in^25^, and placing a few active elements randomly into the IRS to estimate the channel as proposed in^26^, this can help in recovering the full CSI. Consequently, reducing the training overhead, but unfortunately, this raises the system complexity and hardware cost. In order to enhance the signal’s received power, a deep neural network (DNN) that has the ability to learn the mapping from the delivered pilot sequences to the BS optimal beamformer and the IRS controller is proposed in^27^. The effect of optimal phase shift detection on the spectral efficiency is widely illustrated in^28^, adopting the CSI statistical approach. Cascaded non-sparse channel estimation in an IRS-aided multiuser MISO system in^29^ leverages the common link between the BS and IRS. The existing work in^30^ considers a multi-user massive MIMO underlay CR system aided by an IRS. A pilot-based sparse channel estimation method has been investigated. A deep deterministic policy gradient (DDPG) based algorithm that solves the received SNR maximization issue in a multi-user IRS-aided MISO communication system is proposed in^31^, and shows that such an algorithm is able to accomplish a commensurate received SINR and less running time in comparison to the semi-definite relaxation-based methods. Motivated by all the above aspects, this work considers a multi-user mm-wave MIMO underlay CR system assisted by an IRS. A pilot-based channel estimation method has been investigated, where the IRS reflecting elements passively redirect the pilot sequences transmitted by the users to the SBS, such that the SBS can estimate the cascaded CSI. A novel DRL-based algorithm is suggested to solve such a non-convex optimization problem effectively and achieve high performance.

## System model

**Fig. 1:**
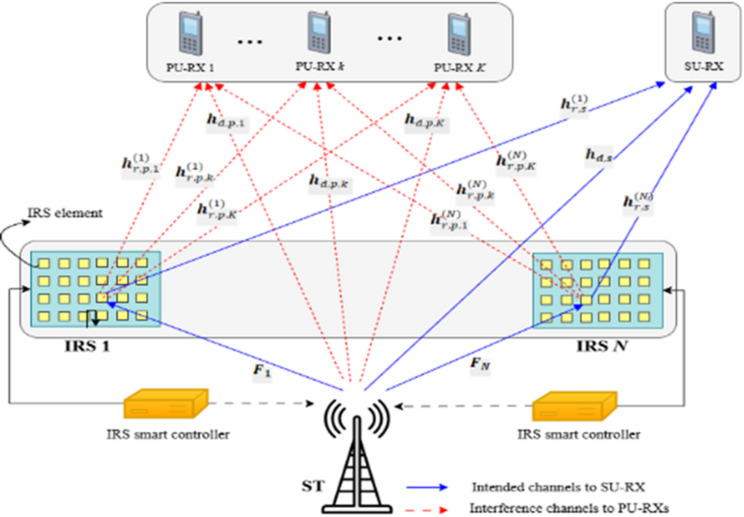
The proposed IRS-assisted CR system.

As illustrated in Fig. 1, an IRS-aided CR downlink network that consists of **N** (*N* ≥ 1) IRSs, a pair of ST and SU-RX, and **K** (K ≥ 1) PU-RXs, is considered. The ST employs **M** transmitting antennas; however, each single RX utilizes only one receiving antenna for simplicity. We presume that the SU utilizes the dedicated frequency band for one or more PUs, and every IRS array consists of **L** passive elements, i.e., (L > 1).

### Channel model

We adopt the concept of block fading channels, which means that the channel coefficients are fixed inside every single block and vary separately among the different blocks. We exploit the channel reciprocity property by adopting the protocol of time division duplex (TDD), and estimate the downlink channels based on the delivered uplink pilot sequences from the PU-RXs and the ST. The ST to PU-RXs and SU-RX baseband corresponding direct-link channel responses are denoted by $\:{\boldsymbol{h}}_{\boldsymbol{d},\boldsymbol{p},\boldsymbol{k}}\in\:{\mathbb{C}}^{M\times\:1}$ for $\:k\in\:\mathcal{K}=\{\mathrm{1,2},3,\dots\:,K\}$ and $\:{\boldsymbol{h}}_{\boldsymbol{d},\boldsymbol{s}}\in\:{\mathbb{C}}^{M\times\:1}$, respectively. All the elements of $\:{\boldsymbol{h}}_{\boldsymbol{d},\boldsymbol{p},\boldsymbol{k}}$ and $\:{\boldsymbol{h}}_{\boldsymbol{d},\boldsymbol{s}}$ represent identically and independent distributed (i.i.d.) complex Gaussian random variables having a zero mean and unit variance, that is, $\:{\boldsymbol{h}}_{\boldsymbol{d},\boldsymbol{p},\boldsymbol{k}}$ ∼ $\:\mathcal{C}\mathcal{N}$($\:{0}_{N}\:$, $\:{\boldsymbol{I}}_{N}$) and $\:{\boldsymbol{h}}_{\boldsymbol{d},\boldsymbol{s}}$ ∼ $\:\mathcal{C}\mathcal{N}$($\:{0}_{N}\:$, $\:{\boldsymbol{I}}_{N}$), respectively. Also, we denote the corresponding channel responses from the ST to each IRS unit by $\:{\boldsymbol{F}}^{\left(\boldsymbol{n}\right)}\in\:{\mathbb{C}}^{L\times\:M}$ for $\:\in\:\mathcal{N}=\{\mathrm{1,2},3,\dots\:,N\}$ .

We can presume that there is a line-of-sight (LoS) path existing in the reflect-link due to the fact that the IRSs are supposed to be placed on the exterior of a building near the users and the BS. AS a result, channel $\:{\boldsymbol{F}}^{\left(\boldsymbol{n}\right)}$ may be expressed by the Rician channel model:

1 $$\:{\:\boldsymbol{F}}^{\left(n\right)}=\sqrt{\frac{{z}_{1}}{{z}_{1}+1}}{\boldsymbol{F}}^{\left(n\right),LoS}+\:\sqrt{\frac{1}{{z}_{1}+1}}{\boldsymbol{F}}^{\left(n\right),NLoS},$$

where $\:{z}_{1}$ denotes the Rician channel constant, $\:{\boldsymbol{F}}^{\left(n\right),LoS}$ $\:\in\:{\mathbb{C}}^{L\times\:M}$ is the LoS channel component, and $\:{\boldsymbol{F}}^{\left(n\right),NLoS}$ $\:\in\:{\mathbb{C}}^{L\times\:M}$ symbolizes the NLoS channel matrix that has multiple i.i.d. complex Gaussian distribution-following elements of a unit variance and zero mean. Furthermore, assuming that both the IRS and the ST are composed of uniform rectangular arrays (URA), the LoS channel components $\:{\boldsymbol{F}}^{\left(\boldsymbol{n}\right),LoS}$ can be modeled, according to the authors in^32^ as follows:

2 $$\:{\boldsymbol{F}}^{\left(\boldsymbol{n}\right),LoS}={\boldsymbol{a}}_{L}\left({\alpha\:}_{AoA}^{\left(n\right)},{\beta\:}_{AoA}^{\left(n\right)}\right){\left({\boldsymbol{a}}_{M}\left({\alpha\:}_{AoD}^{\left(n\right)}\right)\right)}^{\dagger},$$

where $\:{\alpha\:}_{AoA}^{\left(n\right)}$ and $\:{\beta\:}_{AoA}^{\left(n\right)}$ represent the azimuthal and elevation arrival angle (AoA) at the nth IRS, respectively, while $\:{\alpha\:}_{AoD}^{\left(n\right)}$ means the departure angle (AoD) from the nth IRS to the ST. The first part of the equation is the IRS steering vector that is modeled by:

3 $$\:{\boldsymbol{a}}_{L}\left({\alpha\:}_{AoA}^{\left(n\right)},{\beta\:}_{AoA}^{\left(n\right)}\right)={\boldsymbol{a}}_{{L}_{1}}^{\left(v\right)}\left({\alpha\:}_{AoA}^{\left(n\right)},{\beta\:}_{AoA}^{\left(n\right)}\right)\otimes\:{\boldsymbol{a}}_{{L}_{2}}^{\left(h\right)}\left({\alpha\:}_{AoA}^{\left(n\right)},{\beta\:}_{AoA}^{\left(n\right)}\right),$$

where the amount of IRS elements in each row and column is represented by $\:{L}_{1}$ and $\:{L}_{2}$, respectively. While the steering vectors in both horizontal and vertical directions are symbolized by $\:{\boldsymbol{a}}_{{L}_{2}}^{\left(h\right)}$ $\:\in\:{\mathbb{C}}^{{L}_{2}\times\:1}$ and $\:{\boldsymbol{a}}_{{L}_{1}}^{\left(v\right)}$ $\:\in\:{\mathbb{C}}^{{L}_{1}\times\:1}$, respectively. Furthermore, these steering vectors are expressed as follows:

4 $$\:{\left[{\boldsymbol{a}}_{{L}_{1}}^{\left(v\right)}\left({\alpha\:}_{AoA}^{\left(n\right)},{\beta\:}_{AoA}^{\left(n\right)}\right)\right]}_{{l}_{1}}={e}^{j\frac{2({l}_{1}-1)\pi\:d}{\lambda\:}cos\left({\beta\:}_{AoA}^{\left(n\right)}\right)cos\left({\alpha\:}_{AoA}^{\left(n\right)}\right)}\:\:\:\:,\:\forall\:{l}_{1}\in {\mathcal{L}}_{1}\:,$$

5 $$\:{\left[{\boldsymbol{a}}_{{L}_{2}}^{\left(h\right)}\left({\alpha\:}_{AoA}^{\left(n\right)},{\beta\:}_{AoA}^{\left(n\right)}\right)\right]}_{{l}_{2}}={e}^{-j\frac{2({l}_{2}-1)\pi\:d}{\lambda\:}cos\left({\beta\:}_{AoA}^{\left(n\right)}\right)sin\left({\alpha\:}_{AoA}^{\left(n\right)}\right)}\:\:\:,\:\forall\:{l}_{2}\in {\mathcal{L}}_{2}\:,\:$$

with *d* representing the actual distance separating the neighboring IRS elements, and *λ* is the carrier wavelength. To facilitate the estimation of AoA and from past experiences, we set d/λ equal to 1/2. By the same concept, the steering vector for the ST, $\:{\boldsymbol{a}}_{M}^{\:}\left({\alpha\:}_{AoD}^{\:}\right)$ $\:\in\:{\mathbb{C}}^{M\times\:1}$, can be given by:

6 $$\:{\left[{\boldsymbol{a}}_{M}^{\:}\left({\alpha\:}_{AoD}^{\:}\right)\right]}_{m}={e}^{-j\frac{2(m-1)\pi\:d}{\lambda\:}sin\left({\alpha\:}_{AoD}^{\:}\right)},$$

In the same manner, the reflect-link channels from the *n*th IRS unit to the PU-RXs and the SU-RX, $\:{\boldsymbol{h}}_{r,p,k}^{\left(n\right)}\in\:{\mathbb{C}}^{L\times\:1}$ and $\:{\boldsymbol{h}}_{r,s}^{\left(n\right)}\in\:{\mathbb{C}}^{L\times\:1}$, respectively, can be modeled by the following:

7 $$\:{\boldsymbol{h}}_{r,p,k}^{\left(n\right)}=\sqrt{\frac{{z}_{2}}{{z}_{2}+1}}{\boldsymbol{h}}_{r,p,k}^{\left(n\right),LoS}+\:\sqrt{\frac{1}{{z}_{2}+1}}{\boldsymbol{h}}_{r,p,k}^{\left(n\right),NLoS},$$

8 $$\:{\boldsymbol{h}}_{r,s}^{\left(n\right)}=\sqrt{\frac{{z}_{3}}{{z}_{3}+1}}{\boldsymbol{h}}_{r,s}^{\left(n\right),LoS}+\:\sqrt{\frac{1}{{z}_{3}+1}}{\boldsymbol{h}}_{r,s}^{\left(n\right),NLoS},$$

and $\:{z}_{2}$ and $\:{z}_{3}$ represent the Rician factors. While, $\:{\boldsymbol{h}}_{r,p,k}^{\left(n\right),LoS}$**=** $\:{\boldsymbol{a}}_{L}\left({\alpha\:}_{AoD,p,k}^{\left(n\right)},{\beta\:}_{AoD,p,k}^{\left(n\right)}\right)\in\:{\mathbb{C}}^{L\times\:1}$ and $\:{\boldsymbol{h}}_{r,s}^{\left(n\right),LoS}=\:{\boldsymbol{a}}_{L}\left({\alpha\:}_{AoD,s}^{\left(n\right)},{\beta\:}_{AoD,s}^{\left(n\right)}\right)\in\:{\mathbb{C}}^{L\times\:1}$ represents the LoS channel components. Furthermore, $\:{\alpha\:}_{AoD,p,k}^{\left(n\right)}$ and $\:{\beta\:}_{AoD,p,k}^{\left(n\right)}$ denote the azimuthal and the elevational AoD leaving the IRS towards the *k*th PU-RX while $\:{\alpha\:}_{AoD,s}^{\left(n\right)}$ and $\:{\beta\:}_{AoD,s}^{\left(n\right)}$ denote the azimuth and elevational AoD, respectively, from the IRS towards the SU-RX.

### Signal modeling

The received signals by the SU-RX and the *k*th PU-RX, which are symbolized by $\:{y}_{s}$ and $\:{y}_{p,k}$, respectively, can be modeled as:

9a $$\:{y}_{s}=\left({\boldsymbol{h}}_{d,s}^{\dagger}+\:\varphi\:\sum\:_{n=1}^{N}{\boldsymbol{h}}_{r,s}^{\left(n\right),\dagger}{\boldsymbol{\varPhi\:}}^{\left(n\right)}{\boldsymbol{F}}^{\left(n\right)}\right){x}_{s}+{u}_{s}\:,\:$$

9b $$\:{\:y}_{p,k}=\left({\boldsymbol{h}}_{d,p,k}^{\dagger}+\:\varphi\:\sum\:_{n=1}^{N}{\boldsymbol{h}}_{r,p,k}^{\left(n\right),\dagger}{\boldsymbol{\varPhi\:}}^{\left(n\right)}{\boldsymbol{F}}^{\left(n\right)}\right){x}_{s}+{u}_{p,k},$$

where $\:\varphi\:$ represents the relative channel gain for the reflect link channels, and may be named the double fading effect as in^33^, usually $\:\varphi\:\ll\:1$, as the reflect link signals suffer from multiple attenuations. While $\:{\boldsymbol{\varPhi\:}}^{\left(n\right)}$ defines the *n*th IRS reflecting coefficients, $\:{\boldsymbol{\varPhi\:}}^{\left(n\right)}=diag\left({\boldsymbol{\theta\:}}^{\left(n\right)}\right)$ and $\:{\boldsymbol{\theta\:}}^{\left(n\right)}=[{\alpha\:}_{1}^{\left(n\right)}\:{e}^{j{\theta\:}_{1}^{\left(n\right)}},\dots\:,{\alpha\:}_{L}^{\left(n\right)}\:{e}^{j{\theta\:}_{L}^{\left(n\right)}}]$ for $\:\in\:\mathcal{N}=\{1,\dots\:,N\}$, as well, $\:{\alpha\:}_{l}^{\left(n\right)}\:$∈ [0, 1] $\:{\mathrm{a}\mathrm{n}\mathrm{d}\:\theta\:}_{l}^{\left(n\right)}$ ∈ [0, 2π] represents the amplitude gain and phase shifts caused by the *l*th element associated with the *n*th IRS, accordingly. Here, the baseband transmitted signal by the ST is represented by $\:{x}_{s}$ $\:\in\:{\mathbb{C}}^{M\times\:1}$ while the additive white Gaussian noise (AWGN) at both the SU-RX and the *k*th PU-RX is denoted by $\:{u}_{s}\sim\:\:\mathcal{C}\mathcal{N}(0,\:{{\upsigma\:}}^{2})\:$and $\:{u}_{p,k}$ ∼ $\:\mathcal{C}\mathcal{N}(0,\:{{\upsigma\:}}^{2})$, respectively. Moreover, $\:{x}_{s}$= (*w)(s)*, where *w* $\:\in\:{\mathbb{C}}^{M\times\:1}$ denotes the beamforming vector of the dedicated information-symbol *s* ∼ $\:\mathcal{C}\mathcal{N}(0,\:1)$. We consider that, $\:{u}_{s}$ includes the interference contributions from PU-TXs that are actually considered as noise at the SU-RX side. The SU and PU delivered signals in (9a) and (9b) can be rewritten as follows:

10a $$\:{y}_{s}={{\boldsymbol{\theta\:}}^{\dagger}\boldsymbol{H}}_{s}{x}_{s}+{u}_{s},$$

10b $$\:{y}_{p,k}={{\boldsymbol{\theta\:}}^{\dagger}\boldsymbol{H}}_{p,k}{x}_{s}+{u}_{p,k},$$

where $\:{\boldsymbol{\theta\:}}^{\dagger}\triangleq\:\left[{\theta\:}^{\left(1\right)},\:{\theta\:}^{\left(2\right)},{\theta\:}^{\left(3\right)},\dots\:,{\theta\:}^{\left(n\right)},1\right]\in\:{\mathbb{C}}^{1\times\:(NL+1)}$; $\:{\boldsymbol{H}}_{s}$ and $\:{\boldsymbol{H}}_{p,k}$ represent the composite channel components for SU-RX and *k* PU-RXs, accordingly, that can be defined as:

11a $$\:{\boldsymbol{H}}_{s}\triangleq\:\left[\begin{array}{c}\varphi\:diag\left({\boldsymbol{h}}_{r,s}^{\left(1\right),\dagger}\right){\boldsymbol{F}}^{\left(1\right)}\\\:\varphi\:diag\left({\boldsymbol{h}}_{r,s}^{\left(2\right),\dagger}\right){\boldsymbol{F}}^{\left(2\right)}\\\:.\\\:.\\\:.\\\:\varphi\:diag\left({\boldsymbol{h}}_{r,s}^{\left(N\right),\dagger}\right){\boldsymbol{F}}^{\left(N\right)}\\\:{\boldsymbol{h}}_{d,s}^{\dagger}\end{array}\right]\in\:{\mathbb{C}}^{\left(NL+1\right)\times\:M}\:,$$

11b $$\:{\boldsymbol{H}}_{p,k}\triangleq\:\left[\begin{array}{c}\varphi\:diag\left({\boldsymbol{h}}_{r,p,k}^{\left(1\right),\dagger}\right){\boldsymbol{F}}^{\left(1\right)}\\\:\varphi\:diag\left({\boldsymbol{h}}_{r,p,k}^{\left(2\right),\dagger}\right){\boldsymbol{F}}^{\left(2\right)}\\\:.\\\:.\\\:.\\\:\varphi\:diag\left({\boldsymbol{h}}_{r,p,k}^{\left(N\right),\dagger}\right){\boldsymbol{F}}^{\left(N\right)}\\\:{\boldsymbol{h}}_{d,p,k}^{\dagger}\end{array}\right]\in\:{\mathbb{C}}^{\left(NL+1\right)\times\:M}\:,$$

From (10a), we can deduce the achievable SU SNR, which can be written as follows:

12 $$\:SN{R}_{s}={{\Gamma\:}}_{s}=\:\:\frac{E\left[\right|{{{\boldsymbol{\theta\:}}^{\dagger}\boldsymbol{H}}_{s}{x}_{s}\left.\right|}^{2}]}{E\left[{u}_{s}^{2}\right]}=\frac{|{{{\boldsymbol{\theta\:}}^{\dagger}\boldsymbol{H}}_{s}w\left.\right|}^{2}}{{{\upsigma\:}}_{\mathrm{s}}^{2}}\:,$$

where the expectation in the numerator; $\:E\left[\right|{{{\boldsymbol{\theta\:}}^{\dagger}\boldsymbol{H}}_{s}{x}_{s}\left.\right|}^{2}]$, adopts the information symbol *s*, but in the denominator; $\:E\left[{u}_{s}^{2}\right]$, is just taken over the AWGN $\:{u}_{s}$. In the same manner, from (10b), the resulting interference power induced by ST and disturbing each PU-RX, that is widely referred to as the Interference Temperature (IT limit) in CR networks, can be derived as:

13 $$\:I{T}_{p,k}=\:E\left[\right|{{{\boldsymbol{\theta\:}}^{\dagger}\boldsymbol{H}}_{p,k}{x}_{s}\left.\right|}^{2}]\:=\:|{{{\boldsymbol{\theta\:}}^{\dagger}\boldsymbol{H}}_{p,k}w\left.\right|}^{2},$$

Mainly, estimating the channel is essential to jointly optimize the passive reflect coefficients of IRSs and the beamforming vector of the ST. An efficient method that enables the ST to obtain the composite channel matrices $\:{\boldsymbol{H}}_{s}$ and $\:{\boldsymbol{H}}_{p,k}$ rather than estimating the entire individual channels, is to follow the channel estimation protocol that is proposed in^34^.

### Problem formulation

We focus on enhancing and boosting the SU achievable rate, i.e., log (1 + $\:SN{R}_{s}$), by simultaneously adjusting the ST beamforming vector and the IRS reflecting-coefficients vector, namely ***w*** and ***θ***, accordingly. At the same time, we should reduce the IT on the U-RX, i.e., $\:I{T}_{p,k}\:$, and the required transmitting power of ST ($\:{p}_{s}=\:\parallel\:w{\parallel\:}^{2}$). These aspects formulate the following optimization problem:

14a $$\:\left(P1\right)\:\:\:\:\:\:\underset{w,\theta\:}{\mathrm{max}}{\mathrm{log}}_{2}\left(1+\frac{|{{{\boldsymbol{\theta\:}}^{\dagger}\boldsymbol{H}}_{s}w\left.\right|}^{2}}{{{\upsigma\:}}_{\mathrm{s}}^{2}}\right)$$

14b $$\:s.t.\:\:|{{{\boldsymbol{\theta\:}}^{\dagger}\boldsymbol{H}}_{p,k}w\left.\right|}^{2}\:\le\:\:{{\Gamma\:}}_{k}\:\:,\:\:\:\:\:k=1,\dots\:\:,K$$

14c $$\:\parallel\:w{\parallel\:}^{2}\:\le\:{P}_{max}$$

14d $$\:\:{\boldsymbol{\theta\:}}_{\boldsymbol{i}}^{\:}\in\:\left[\mathrm{0,2}\pi\:\right]\:\:\:\:\:,\forall\:i\in\:\mathcal{L}$$

where $\:\mathcal{L}=\{1,\dots\:,NL+1\}$. According to problem (P1), (14a) maximizes the SU achievable rate; (14b) represents the IT limit with a threshold $\:{{\Gamma\:}}_{k}$ for *k* PU-RXs; while the ST required power allocation constraint is represented by (14c), and (14d) is the passive IRS-phase shifts.

Unfortunately, this is a non-convex optimization problem since the objective function is completely non-concave over the joined ***w*** and ***θ***, due to the monotonically increasing logarithmic term. Consequently, it is difficult to be addressed by regular convex optimization methods. To tackle such a problem, there are two main directions of solution suggested in recent years. One of them is to decouple $\:\left(P1\right)\:$ into two subproblems by means of the alternating optimization (AO) based algorithm. When one of ***w*** and ***θ*** is fixed, the resultant problem can be simply solved. Thus, $\:\left(P1\right)\:$ can be sub-optimally solved by iteratively optimizing one of ***w*** and ***θ*** and keeping the other fixed at each iteration until reaching convergence. Block coordinate descent (BCD) and semidefinite relaxation (SDR) based algorithms can be used for this purpose^35^. However, raising the size of the IRS array significantly necessitates rerunning such optimization approaches anytime the system parameters are altered, which requires high computational complexity and complicated mathematical transformations. Another way to solve $\:\left(P1\right)$ By benefiting from the deep reinforcement learning (DRL) techniques, a deep deterministic policy gradient (DDPG) based algorithm was suggested in^31^ to solve a received SNR maximization issue through a single-user MIMO system aided by IRS, and it has precisely achieved a comparable performance with obviously less time and high learning efficiency, in comparison to the SDR-based method. So, in this paper, we aim to efficiently solve $\:\left(P1\right)$ utilizing the DRL approaches, specifically the soft actor-critic (SAC)-based algorithms.

## SAC-based algorithm

We devote this part to introduce a SAC-based algorithm that efficiently handles the problem $\:\left(P1\right)$. However, it is necessary to first understand the working method of DL and DRL.

### Introduction to DRL

Machine learning (ML) can be defined as the process of gleaning valuable information from an available data set. The main purpose of machine learning algorithms is to determine a mathematical formula that results in suitable solutions to a predefined practical problem when they are provided with accurate inputs. The desired mathematical formula is established by defining the system’s output as a function of several inputs that are called training data. According to this knowledge, the algorithm can make accurate decisions on the expected output when it is fed with any new input. As in^5^, based on the type of data available, ML approaches can be classified into three main branches: Supervised Learning, Unsupervised Learning, and Reinforcement Learning(RL). In this paper, we focus on RL systems, in which the algorithm is utilized to recognize the environment state and then execute the required actions, keeping in mind that each action results in a distinct reward. The algorithm has a target to learn an optimal policy that uses the states as inputs to determine the optimal action or system output^36^. To increase the ability of deciding which action to execute in order to increase the accumulated reward, the algorithm relies on the cooperation between the RL agent and the surrounding environment represented by the state (s), the action (a), the reward (r), the policy (π), and the next state.

RL evaluates the efficiency of a certain policy (π) using a Q factor; $\:{q}_{{\uppi\:}}(s,a)$, specifying the comprehensive cumulative reward that the agent accomplishes if it begins from a state (s) and executes an action (a). However, in the case of a continuous action space, it is more difficult to obtain the Q value using the traditional tabular solution methods. DRL uses a parameterized function to obtain the Q factor, adopting the concept of function approximation that is performed by using a specified deep neural network (DNN)^31^.

### Basics of the SAC-proposed algorithm

Firstly, we should explain the relative entropy concept, or Kullback-Leibler (KL) divergence, which was proposed in^37^, it describes the degree of similarity for two distinct distributions. A small entropy value indicates greater similarity between the considered distributions. If $\:p$ and $\:q$ represent two continuous probability distributions with a random variable $\:\mathcal{z}$ from the set $\:\mathcal{Z}$, the KL divergence can be represented by:

15 $$\:{\mathrm{D}}_{\mathrm{K}\mathrm{L}}\left(\mathrm{p}\|\mathrm{q}\:\right)=\:{\int\:}_{\mathcal{Z}}^{\:}\mathrm{p}\left(\mathrm{z}\right)\mathrm{log}\left(\frac{\mathrm{p}\left(\mathrm{z}\right)}{\mathrm{q}\left(\mathrm{z}\right)}\right)\mathrm{d}\mathrm{z},\:\:$$

where $\:p\left(z\right)$ and $\:q\left(z\right)$ represent the probability density functions of $\:p$ and $\:q$. To enable the SAC agent to benefit from the environment interactions, an experience (storage) replay $\:\mathcal{P}$ with a predetermined size should be constructed. For a time step (t), the SAC agent executes an action $\:{a}_{t}$ based on a state $\:{s}_{t}$ acquiring a reward $\:{r}_{t}$ and finally reaches the next-state $\:{s}_{t+1}$ producing a transition tuple ($\:{s}_{t}$, $\:{a}_{t}$, $\:{r}_{t}$, $\:{s}_{t+1}$) that is simultaneously placed into the predefined storage replay $\:\mathcal{P}$.

The SAC learning procedure involves two main stages, which are known as policy evaluation and improvement stages. The DNN parameters are modified while these two stages are performed successively. During the first stage, the SAC algorithm tries to enhance the soft $\:{Q}_{\:}$value, that is defined by:

16 $$\:{q}_{\pi\:}^{soft}\left({s}_{t},\:{a}_{t}\right)={r}_{t}+\:\gamma\:{\mathbb{E}}_{{s}_{t+1}\sim p}\left[{v}_{\pi\:}^{soft}\left({s}_{t+1}\right)\right],$$

where the soft state value; $\:{v}_{\pi\:}^{soft}\left({s}_{t}\right)$ is defined as:

17 $$\:{\:\:v}_{\pi\:}^{soft}\left({s}_{t}\right)={\mathbb{E}}_{{a}_{t}\sim\pi\:}\left[{q}_{\pi\:}^{soft}\left({s}_{t},\:{a}_{t}\right)-\lambda\:\mathrm{log}(\pi\:\left({a}_{t}|{s}_{t}\right)\right],$$

In (16) and (17), the state-transition probability marginals are represented by $\:p$, while λ indicates a factor that is used to adjust the value of the logarithmic term and $\:\pi\:\left({a}_{t}|{s}_{t}\right)$ defines a stochastic policy for action selection based on a specified state $\:{s}_{t}.$

During the second stage, the improved SAC policy $\:{{\uppi\:}}_{new}$ is used to enhance the soft $\:{Q}_{\:}$value in addition to keeping both policy and $\:{Q}_{\:}$value distributions as close as possible. Thus, the new policy $\:{{\uppi\:}}_{new}$ is usually maintained by reducing the relative entropy of the policy distribution $\:{\pi\:}^{{\prime\:}}\left(.|{s}_{t}\right)$ and the *Q* factor distribution $\:{Q}_{\pi\:}^{soft}\left({s}_{t},\:.\right)$as much as possible.

18 $$\:{{\uppi\:}}_{\mathrm{n}\mathrm{e}\mathrm{w}}=\mathrm{arg}\underset{{{\uppi\:}}^{{\prime\:}}\in\:{\Pi\:}}{\mathrm{min}}{\mathrm{D}}_{\mathrm{K}\mathrm{L}}\left({{\uppi\:}}^{{\prime\:}}\left(.\left|{\mathrm{s}}_{\mathrm{t}}\right.\right)\|\frac{\mathrm{exp}\left(\frac{1}{{\uplambda\:}}{\mathrm{Q}}_{{\uppi\:}}^{\mathrm{s}\mathrm{o}\mathrm{f}\mathrm{t}}\left({\mathrm{s}}_{\mathrm{t}},\:.\right)\right)}{{Z}_{{\uppi\:}}\left({\mathrm{s}}_{\mathrm{t}}\right)}\right),$$

where $\:{{\uppi\:}}^{{\prime\:}}$ usually selected over a flexible policy group Π, and $\:{Z}_{{\uppi\:}}\left({\mathrm{s}}_{\mathrm{t}}\right)\:$represents a partition function that is commonly used for distribution normalization^38^.

### Algorithm description

**Fig. 2:**
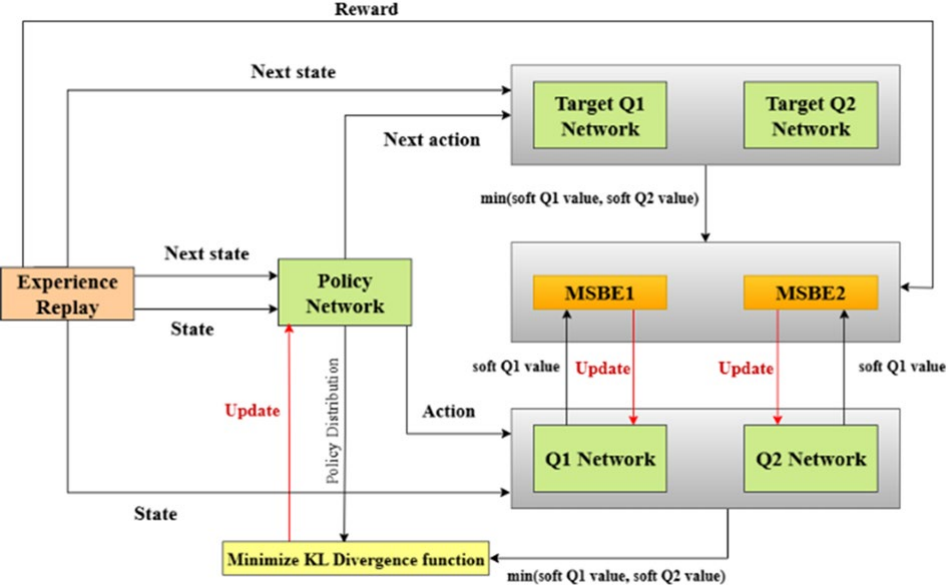
The framework of the proposed SAC algorithm.

Figure 2 illustrates the framework of the proposed SAC-based algorithm, which consists of five DNNs, i.e., Q1, Q2, policy, target Q1, and target Q2 networks with corresponding parameters $\:{\phi\:}_{{q}_{1}}$, $\:{\phi\:}_{{q}_{2}}$, $\:{\phi\:}_{{\upmu\:}}$, $\:{\phi\:}_{{q}_{1}^{{\prime\:}}}$, and $\:{\phi\:}_{{q}_{2}^{{\prime\:}}}$, respectively. If the input is represented by a state $\:{s}_{t}$ and an action $\:{a}_{t}$, then, the Q1 and Q2 networks provide outputs as soft Q1 and Q2 values. Considering the following state $\:{s}_{t+1}$ and the action $\:{a}_{t+1}$ as input, causes the target networks to produce new soft Q1 and Q2 values. Moreover, feeding an input $\:{s}_{t}$ to the policy(main) network yields a policy (π) that represents a Gaussian action distribution, parameterized by mean ζ and standard deviation σ. Subsequently, an optimized action $\:{a}_{t}$ can be selected from this action-policy distribution, i.e.,

19 $$\:\:\:{a}_{t}\sim\:\pi\:\left({a}_{t}|{s}_{t};{\phi\:}_{{\upmu\:}};{\upzeta\:};{\upsigma\:}\right),$$

In the training process, taking a sample from the experience replay $\:\mathcal{P}$ yields in obtaining a *k*th transition tuple ($\:{s}_{k}$, $\:{a}_{k}$, $\:{r}_{k}$, $\:{s}_{k+1}$). With the state $\:{s}_{k+1}$ fed to the main network, it generates a policy distribution π, which is sampled to obtain the action $\:{a}_{k+1}$ as mentioned before. Feeding in a state $\:{s}_{k}$ and an action $\:{a}_{k}$ as inputs to the Q1 and Q2 networks, this provides soft Q1 and Q2 values$\:,\:{q}_{\pi\:}^{soft}\left({s}_{k},\:{a}_{k};{\phi\:}_{{q}_{1}}\right)$ and $\:{q}_{\pi\:}^{soft}\left({s}_{k},\:{a}_{k};{\phi\:}_{{q}_{2}}\right)$, respectively. Similarly, a state $\:{s}_{k+1}$ and an action $\:{a}_{k+1}$ make the target networks provide soft Q1 and Q2 values, $\:{q}_{\pi\:}^{soft}\left({s}_{k+1},\:{a}_{k+1};{\phi\:}_{{q}_{1}^{{\prime\:}}}^{\:}\right)\:$and $\:{q}_{\pi\:}^{soft}\left({s}_{k+1},\:{a}_{k+1};{\phi\:}_{{q}_{2}^{{\prime\:}}}^{\:}\right)$. Letting,

20 $$\:q=\:\underset{\:}{\mathrm{min}}\left({q}_{\pi\:}^{soft}\left({s}_{k},\:{a}_{k};{\phi\:}_{{q}_{1}}\right){,q}_{\pi\:}^{soft}\left({s}_{k},\:{a}_{k};{\phi\:}_{{q}_{2}}\right)\right),$$

21 $$\:{q}^{{\prime\:}}=\:\underset{\:}{\mathrm{min}}\begin{array}{c}\left({q}_{\pi\:}^{soft}\left({s}_{k+1},\:{a}_{k+1};{\phi\:}_{{q}_{1}^{{\prime\:}}}^{\:}\right){,q}_{\pi\:}^{soft}\left({s}_{k+1},\:{a}_{k+1};{\phi\:}_{{q}_{2}^{{\prime\:}}}^{\:}\right)\right),\:\\\:\end{array}\:$$

Updating the Q1 network parameters adopts eliminating the mean-squared Bellman error (MSBE1), which depends on $\:{r}_{k}$, $\:{q}^{{\prime\:}}$, and $\:{q}_{\pi\:}^{soft}\left({s}_{k},\:{a}_{k};{\phi\:}_{{q}_{1}}\right)$. Similarly, minimizing the MSBE2 will update the Q2 network parameters depending on $\:{r}_{k}$, $\:{q}^{{\prime\:}}$, and $\:{q}_{\pi\:}^{soft}\left({s}_{k},\:{a}_{k};{\phi\:}_{{q}_{2}}\right)$. However, the policy network parameters are modified by reducing the relative entropy described by Eq. (18) as much as possible. Also, we should update the temperature λ to control the stochasticity of the generated policy. Finally, the soft update method is utilized to update the $\:{\phi\:}_{{q}_{1}^{{\prime\:}}}^{\:}$ and $\:{\phi\:}_{{q}_{2}^{{\prime\:}}}^{\:}$ parameters as will be shown in the next subsections.

Conforming to (19), an action $\:{a}_{t}$ should be selected from an action distribution, which causes a complexity in training the entire DNNs through back-propagation, as sampling is not differential. A reparameterization trick, which is proposed in^40^, guarantees that all equations utilized in training the DNNs are actually differential. Consequently, the action $\:{a}_{k}$ during this stage can be recovered by adopting the Hadamard product concept:

22 $$\:{\boldsymbol{a}}_{\boldsymbol{k}}=\:{\boldsymbol{\upzeta\:}}_{\mathbf{k}}+\:{\boldsymbol{\upsigma\:}}_{\mathbf{k}}\odot\:{\varepsilon\:},$$

where$\:\:{{\upzeta\:}}_{\mathrm{k}}$ and $\:{{\upsigma\:}}_{\mathrm{k}}$ are the outputs provided by the policy network when it is fed with an input $\:{s}_{k}$. While ε is usually selected over a typical regular distribution similar to the action distribution.

We adopt a reward-impact adjustment approach for the proposed SAC-based algorithm to enhance the learning efficiency and consequently the stability. The suggested approach mainly adjusts the reward-impact on the target Q value by multiplying the reward by an appropriate factor during the learning process to provide the DRL agent with more accurate guidance. The SAC learning procedure involves the policy evaluation and improvement stages. The DNN parameters are updated while these two stages are performed successively. At a time step t, the reward $\:{r}_{t}\in\:\mathbb{R}$ is actually equivalent to the SU rate $\:{{\mathrm{R}}_{\mathrm{s}}={\mathrm{log}}_{2}(1+{\Gamma\:}}_{s})$ if and only if the interference contributions at PU-RX do not exceed the predefined interference temperature (IT) limit, which only occurs after a sufficient training stage with a suitable adjustment factor, as explained by details in the next part.

### Training of DNNs

This section illustrates the process of training the five DNNs and the adaptive updating of the $\:\lambda\:$ factor to enhance the algorithm’s acquired policy.

*1) Q networks training*:

According to (16) and (17), sampling an $\:{N}_{B}$ batch-size from the storage replay $\:\mathcal{P}$, the action $\:{a}_{k+1}$ caused by a state $\:{s}_{k+1}$ is directly obtained. Thus, $\:{a}_{k+1}\sim\:\pi\:\left({a}_{k+1}|{s}_{k+1};{\phi\:}_{{\upmu\:}}\right)$, and the soft target Q factor related to this tuple, symbolized by $\:{y}_{k}$, is given by:

23 $$\:{y}_{k}^{soft}=\:\eta\:{r}_{k}+\:\gamma\:\left[\underset{i=\mathrm{1,2}}{\mathrm{min}}{q}_{\pi\:}^{soft}\left({s}_{k+1},\:{a}_{k+1};{\phi\:}_{{q}_{1}^{{\prime\:}}}^{\:}\right)-\:\lambda\:\mathrm{log}(\pi\:\left({a}_{k+1}|{s}_{k+1};{\phi\:}_{{\upmu\:}}\right)\right],$$

whereas $\:\eta\:$ is a suitable factor used to adapt the effect of the reward $\:{r}_{k}$ on $\:{y}_{k}^{soft}$, and the discount rate is specified by γ ∈ [0, 1]. The (min) operation in (23) determines the smallest value of those generated by the two target Q networks to prevent overestimation, which is referred to as the clipped double-Q trick, which is illustrated in^39^. The MSBE1 between the $\:{y}_{k}^{soft}$ and the soft Q value provided by the Q1 network $\:{q}_{\pi\:}^{soft}\left({s}_{k},\:{a}_{k};{\phi\:}_{{q}_{1}}\right)$, is represented by $\:{L(\phi\:}_{{q}_{1}})$ where:

24a $$\:{L(\phi\:}_{{q}_{1}})=\frac{1}{{N}_{B}}{\sum\:_{k=1}^{{N}_{B}}\left({y}_{k}^{soft}-\:{q}_{\pi\:}^{soft}\left({s}_{k},\:{a}_{k};{\phi\:}_{{q}_{1}}\right)\right)}^{2},$$

In the same manner, the MSBE2 between the $\:{y}_{k}^{soft}$ and the soft Q value provided by the Q2 network $\:{q}_{\pi\:}^{soft}\left({s}_{k},\:{a}_{k};{\phi\:}_{{q}_{2}}\right)$, is represented by $\:{L(\phi\:}_{{q}_{2}})$ where:

24b $$\:\:{L(\phi\:}_{{q}_{2}})=\frac{1}{{N}_{B}}{\sum\:_{k=1}^{{N}_{B}}\left({y}_{k}^{soft}-\:{q}_{\pi\:}^{soft}\left({s}_{k},\:{a}_{k};{\phi\:}_{{q}_{2}}\right)\right)}^{2},$$

Based on the learning rate$\:\:\beta\:$ and the gradients of$\:\:{L(\phi\:}_{{q}_{1}})\:$and $\:\:{L(\phi\:}_{{q}_{2}})$, the parameters of the two *Q* networks are adjusted as follows:

25a $$\:{\phi\:}_{{q}_{1}}=\:{\phi\:}_{{q}_{1}}-\:\beta\:{\nabla\:}_{{\phi\:}_{{q}_{1}}}{L(\phi\:}_{{q}_{1}}),$$

25b $$\:{\phi\:}_{{q}_{2}}=\:{\phi\:}_{{q}_{2}}-\:\beta\:{\nabla\:}_{{\phi\:}_{{q}_{2}}}{L(\phi\:}_{{q}_{2}}),$$

*2) The policy network training*:

Based on Eqs. (15) and (18), with the *k*th tuple ($\:{s}_{k},\:{a}_{k}$) given, the relative entropy between the soft Q value distribution $\:{Q}_{\pi\:}^{soft}\left({s}_{k},\:.;{\phi\:}_{{q}_{i}}\right)$ output from the $\:{Q}_{i}\:$network and the policy distribution $\:{\pi\:}^{\:}\left(.|{s}_{k};{\phi\:}_{{\upmu\:}}\right)$, represented by $\:{d}_{i}\left({\phi\:}_{{\upmu\:}}\right)$, is given by:

26 $$\begin{aligned}&{d}_{i}\left({\phi\:}_{{\upmu\:}}\right)=\:{\mathrm{D}}_{\mathrm{K}\mathrm{L}}\left({\pi\:}^{\:}\left(.|{s}_{k};{\phi\:}_{{\upmu\:}}\right)\|\frac{\mathrm{exp}\left(\frac{1}{{\uplambda\:}}{Q}_{\pi\:}^{soft}\left({s}_{k},\:.;{\phi\:}_{{q}_{i}}\right)\right)}{{Z}_{\pi\:}\left({\mathrm{s}}_{\mathrm{k}}\right)}\right)\:\nonumber\\&={\mathbb{E}}_{{(s}_{k},{a}_{k})\sim\mathcal{D}}\left[{\lambda\:\mathrm{log}({\pi\:}^{\:}\left({a}_{k}|{s}_{k};{\phi\:}_{{\upmu\:}}\right)-\:q}_{\pi\:}^{soft}\left({s}_{k},\:{a}_{k};{\phi\:}_{{q}_{i}}\right)\:\right]\:,\:i=\mathrm{1,2}.\end{aligned}$$

Notice that the constant term $\:{\mathrm{Z}}_{{\uppi\:}}\left({\mathrm{s}}_{\mathrm{k}}\right)$ does not exist in the second equality of (26) since it is eliminated by the derivative operation in the process of updating the policy network parameters. Making good use of the clipped-Q trick and broadening the normal expression with respect to $\:\mathcal{P}$, the relative entropy $\:d\left({\phi\:}_{{\upmu\:}}\right)$for the policy network training may be defined as:

27 $$\:d\left({\phi\:}_{{\upmu\:}}\right)=\:\underset{\:}{\mathrm{min}}\left({d}_{1}\left({\phi\:}_{{\upmu\:}}\right),\:{d}_{2}\left({\phi\:}_{{\upmu\:}}\right)\right)\:=\frac{1}{{N}_{B}}{\sum\:_{k=1}^{{N}_{B}}\left({\lambda\:\mathrm{log}({\pi\:}^{\:}\left({a}_{k}|{s}_{k};{\phi\:}_{{\upmu\:}}\right)-\:q}_{\pi\:}^{soft}\left({s}_{k},\:{a}_{k};{\phi\:}_{{q}_{i}}\right)\right)\:,}^{\:}$$

Based on the learning rate$\:\:{\beta\:}^{{\prime\:}}$ and the gradient of $\:\:d\left({\phi\:}_{{\upmu\:}}\right)$, the policy network parameter, $\:{\phi\:}_{{\upmu\:}}\:$, can be updated as follows:

28 $$\:\:\:\:\:\:\:\:\:\:\:\:\:\:\:\:{\phi\:}_{{\upmu\:}}=\:{\phi\:}_{{\upmu\:}}-\:{\beta\:}^{{\prime\:}}{\nabla\:}_{{\phi\:}_{{\upmu\:}}}d\left({\phi\:}_{{\upmu\:}}\right),$$

*3) Target Q networks training*:

The double target Q networks parameters; $\:{\phi\:}_{{q}_{1}^{{\prime\:}}}^{\:}$ and $\:{\phi\:}_{{q}_{2}^{{\prime\:}}}^{\:}$respectively, can be updated using the soft update method proposed in^39^ to guarantee a stable training, and can be represented by:

29a $$\:{\phi\:}_{{q}_{1}^{{\prime\:}}}^{\:}=\:{\tau\:}_{{\phi\:}_{{q}_{1}}}+\left(1-\tau\:\right){\phi\:}_{{q}_{1}^{{\prime\:}}}^{\:}\:\:\:,\:\:\:0<\tau\:\ll\:1,$$

29b $$\:{\phi\:}_{{q}_{2}^{{\prime\:}}}^{\:}=\:{\tau\:}_{{\phi\:}_{{q}_{2}}}+\left(1-\tau\:\right){\phi\:}_{{q}_{2}^{{\prime\:}}}^{\:}\:\:\:,\:\:\:0<\tau\:\ll\:1,$$

4) Temperature λ updating

It is employed to control the value of the entropy logarithmic term in Eq. (17). During the early learning stage, the action space is usually not sufficiently explored. So, the temperature λ is raised to let the SAC agent explore more actions. Furthermore, once the SAC agent has adequately explored the action space, the temperature factor λ may be decreased. Thus, it is essential to adjust the temperature λ adaptively. To achieve that, firstly, the cumulative reward ($\:{r}_{t}$) optimization problem, that adjusts the algorithm’s policy for each time step in the range of [0, T], subject to the minimal entropy threshold, is considered. This maximization problem is defined as follows:

30a $$\:\:\left(\mathrm{P}2\right):\underset{{\pi\:}_{0},...,\:{\pi\:}_{T}}{\mathrm{max}}{\mathbb{E}}_{{\rho\:}^{\pi\:}}\:\left[\sum\:_{t=0}^{T}{r}_{t}\right]\:\:,\:\:$$

30b $$\:s.t\:\:\:\:\:{\mathbb{E}}_{{({s}_{t},a}_{t})\sim{\rho\:}^{\pi\:}}\left[-\:\lambda\:\mathrm{log}(\pi\:\left({a}_{t}|{s}_{t}\right)\right]\ge\:{H}_{0}\:,\:t=\mathrm{0,1},\dots\:,T,$$

where $\:{\rho\:}^{\pi\:}$ represents the state-to-action policy-induced distribution, and $\:{H}_{0}\:$specifies a predetermined minimal entropy limit. Assuming that, all $\:{({s}_{t},a}_{t})$ tuples utilized in the remaining formulations in this subsection conform the $\:{\rho\:}^{\pi\:}$ distribution. Then, in accordance with the concept of Markov Decision Process (MDP), (P2) may be extended by using the bootstrapping concept as in^40^ and solved iteratively^41^.

### The overall proposed algorithm

The construction process of the state, action, and reward of the suggested SAC approach is comprehensively illustrated as follows:

*1) The state*:

The proposed state must encompass all the variables that govern the expected action, i.e., the phase shift of the IRS reflecting elements, in addition to the prior action. Hence, at a time step t, the state $\:{s}_{t}$ consists of the CSI associated with the entire communication links, the *(t – 1)* IRS phase shifts, and finally the ST transmit power at the same step, symbolized by $\:\boldsymbol{G}$, $\:{\boldsymbol{\Omega\:}}^{t-1}$, and $\:{\boldsymbol{p}}_{s}^{t-1}$ respectively, with

31 $$\:\:\boldsymbol{G}\triangleq\:\:\left[{\boldsymbol{H}}_{s}\:,\:{\boldsymbol{H}}_{p,k}\right]\:,$$

32 $$\:{\boldsymbol{\Omega\:}}^{t-1}\triangleq\:\left[{\theta\:}_{{1}_{1}}^{t-1},{\theta\:}_{{1}_{n}}^{t-1},\:\dots\:,{\theta\:}_{{1}_{L}}^{t-1},\:{\theta\:}_{{N}_{1}}^{t-1},{\theta\:}_{{N}_{n}}^{t-1},\:\dots\:,{\theta\:}_{{N}_{L}}^{t-1}\:\right]\:,$$

Here, $\:{\theta\:}_{{N}_{l}}^{t-1}$ represents the *l*th reflecting element’s phase shift for each utilized IRS array. Consequently, the state $\:{s}_{t}$ can be defined mathematically as:

33 $$\:{s}_{t}=\left[\boldsymbol{G},\:{\boldsymbol{\Omega\:}}^{t-1},\:{\boldsymbol{p}}_{s}^{t-1}\right],$$

*2) The action*:

From problem (P1), it is observed that the optimal transmit power, $\:{p}_{s}^{\:}$, with a given $\:\theta\:$ may be expressed in closed-form as:

34 $$\:\:{p}_{s}^{*}=\mathrm{max}\left(0,\mathrm{min}\left(\frac{\frac{|{{{\boldsymbol{\theta\:}}^{\dagger}\boldsymbol{H}}_{p,k}w\left.\right|}^{2}-{{\upsigma\:}}_{\mathrm{s}}^{2}}{{{\Gamma\:}}_{k}}}{|{{{\boldsymbol{\theta\:}}^{\dagger}\boldsymbol{H}}_{s}w\left.\right|}^{2}},{P}_{max}\right)\right),$$

Therefore, the action $\:{a}_{t}$ may be mainly generated depending on the RIS phase shifts of each element in the RIS units, and it is directly obtained according to the output action distribution formed by the main network using Eq. (19) $\:\Rightarrow\:{a}_{t}\sim\:\pi\:\left({a}_{t}|{s}_{t};{\phi\:}_{{\upmu\:}};{\upzeta\:};{\upsigma\:}\right).$

*3) The reward*:

At time step t, the reward $\:{r}_{t}\in\:\mathbb{R}$ is actually equivalent to the SU rate $\:{{\mathrm{R}}_{\mathrm{s}}={\mathrm{log}}_{2}(1+{\Gamma\:}}_{s})$ if and only if the interference contributions at PU-RX do not exceed the predefined IT limit, and $\:{{\Gamma\:}}_{s}$ is calculated by equation (12) and denotes the received SNR at the SU-RX side.

All aspects of the proposed SAC algorithm are summarized in Algorithm 1. The optimized IRS reflecting phase shift vector;$\:\left[{\boldsymbol{\theta\:}}_{1}^{\left(1\right)},{\boldsymbol{\theta\:}}_{2}^{\left(1\right)},\:\dots\:,{\boldsymbol{\theta\:}}_{\boldsymbol{L}}^{\left(1\right)},\:{\boldsymbol{\theta\:}}_{1}^{\left(\boldsymbol{N}\right)},{\boldsymbol{\theta\:}}_{2}^{\left(\boldsymbol{N}\right)},\:\dots\:,{\boldsymbol{\theta\:}}_{\boldsymbol{L}}^{\left(\boldsymbol{N}\right)}\:\right]$, is obtained by the output of Algorithm 1; $\:{a}^{*}$, while the required ST’s transmit power $\:{p}_{s}^{\:}$ is determined by the equation (34).

**Figure:**
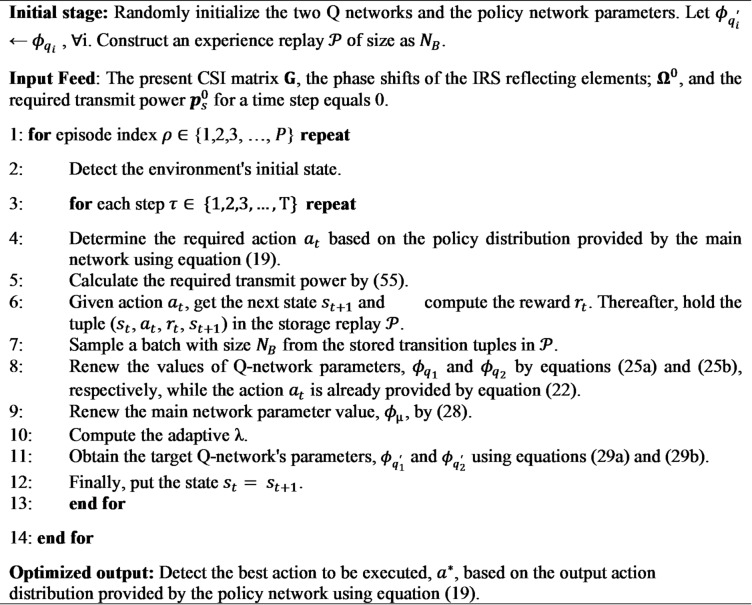
**Algorithm 1** The proposed SAC Algorithm.

## Antenna array design

In this section, we propose a wideband array composed of 16 antenna elements that operates between 3 and 7 GHz for the ST base station. The array elements are positioned in a constrained area and printed on a square-shaped substrate. Good polarization diversity can be attained by isolating the antenna elements well. The proposed ST array is manufactured, tested, and simulated. The representative outcomes, impedance matching (S- parameters), and array total efficiency are examined. Additionally, a significantly small envelope correlation coefficient, i.e., ECC less than 0.02, and an excellent array efficiency of roughly 90% are obtained to validate the MIMO performances.

### Array structure

**Fig. 3:**
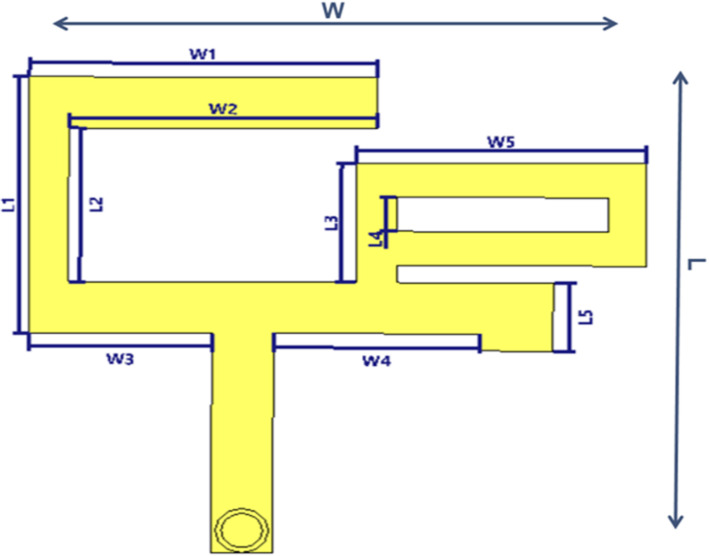
The configuration of the ST single antenna element.

The BS antenna single element is a conventional multi-branch monopole, fed by a 50 microstrip line as shown in Fig. 3. An operating range from 3 to 6.5 GHz can be covered by coupling the resonant modes of the various radiators. The proposed antenna has enough bandwidth to support MIMO operation in the majority of 5G spectrum, encompassing the LTE and the new radio frequency bands. Comparing the suggested array to the traditional printed monopole antenna, the suggested antenna array achieves a more compact size.

A 16-element ST base station MIMO array can be properly formed. The square-shaped system substrate is made of FR4 material, with a relative permittivity of 4.4 and a loss tangent of 0.02.

**Fig. 4:**
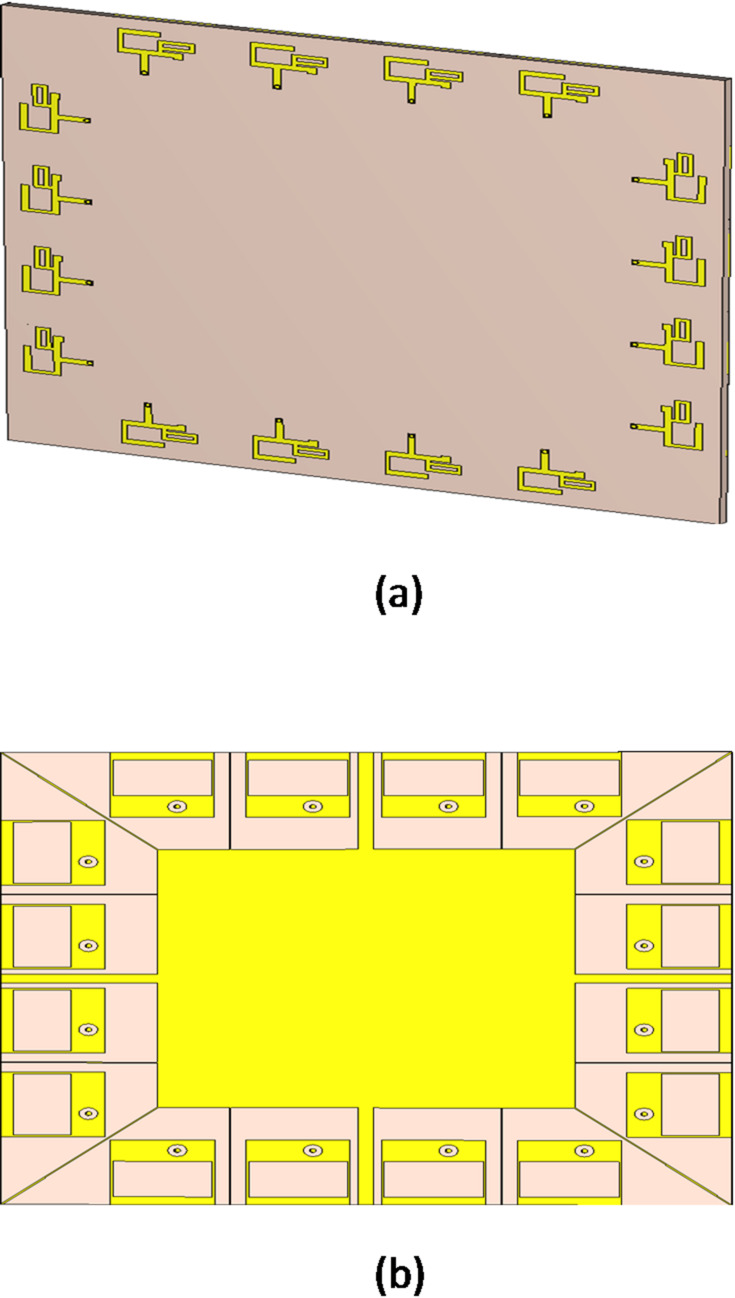
The structure of the suggested 16 element ST array. (**a**) Front surface and (**b**) The substrate’s back surface.

The FR4 substrate is 140 mm ×140 mm × 1.5 mm in size. On the front surface of the substrate, the identical ST array elements are arranged as shown in Fig. 4a, four BS antennas are printed at each side of the square, with an 11 mm distance between each pair of adjacent antenna elements. With a ground plane that is made up of rectangular slots having the measurements 19 mm x 11 mm, and printed on the substrate’s back surface, as shown in Fig. 4b.

### Simulated S-parameters

**Fig. 5:**
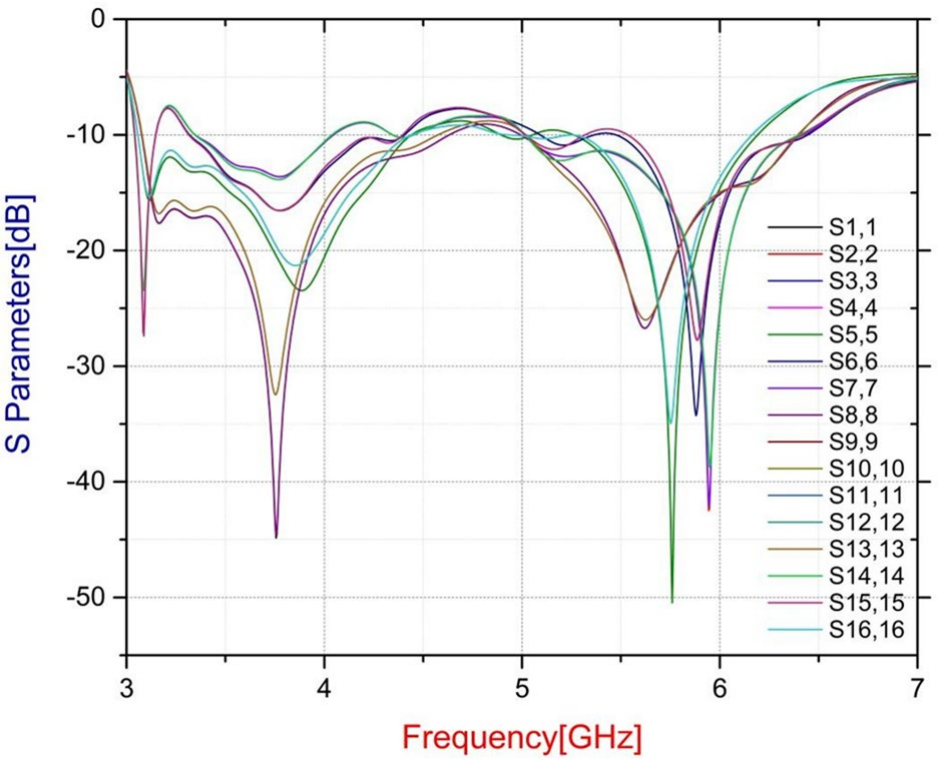
Simulated S-parameters.

Figure 5 displays the simulated return losses, i.e., S-parameters for the suggested ST array. Since the array structure is symmetric, only the reflection coefficients for elements 1–4 are shown; this makes up 25% of the entire array. Thus, over the operating bandwidth of 3–7 GHz, the proposed MIMO array maintains satisfactory impedance matching with acceptable return losses below - 6 dB.

### Antenna fabrication

**Fig. 6:**
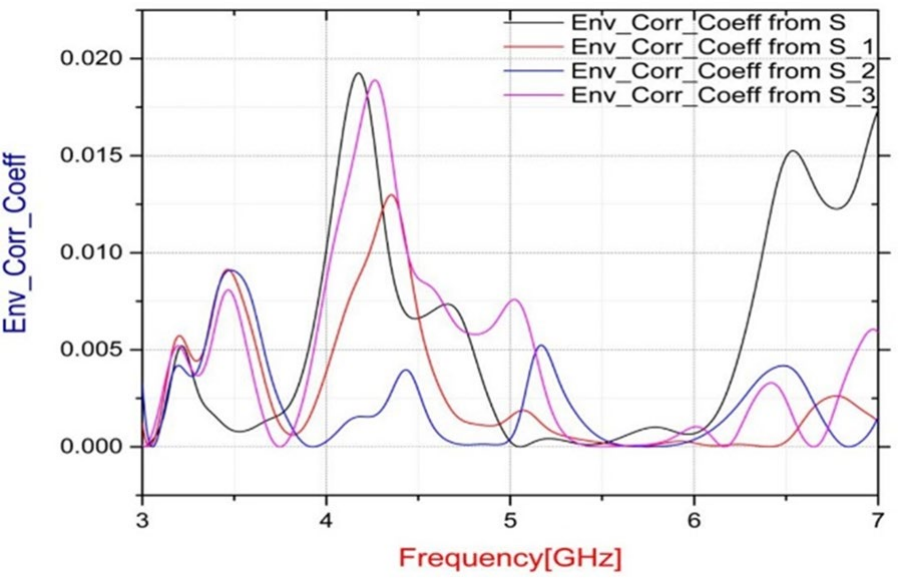
The simulated ECC.

A 16-element ST MIMO array prototype has been practically fabricated to check the feasibility of the suggested. The Envelope Correlation Coefficient (ECC), which measures the similarity degree of the received signals by two different antenna elements, is investigated to further demonstrate the performance of the suggested array. Considering an isotropic multipath environment with respect to both power density and polarizations to simplify the computation of the ECC value. Figure 6 displays the ECC range of the suggested ST array, where the ECC values are evaluated based on the antennas’ far-field radiation obtained from CST for every two neighboring elements. As shown in the figure, the maximum ECC obtained is below 0.02 over the entire operating bandwidth, indicating excellent MIMO performance.

**Fig. 7:**
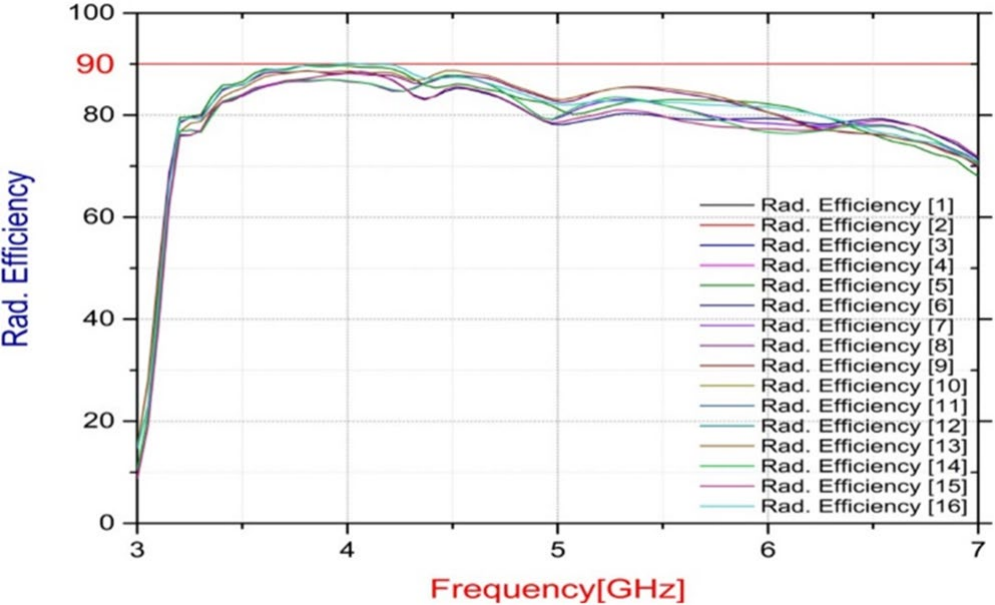
The radiation efficiency of the suggested array.

**Fig. 8:**
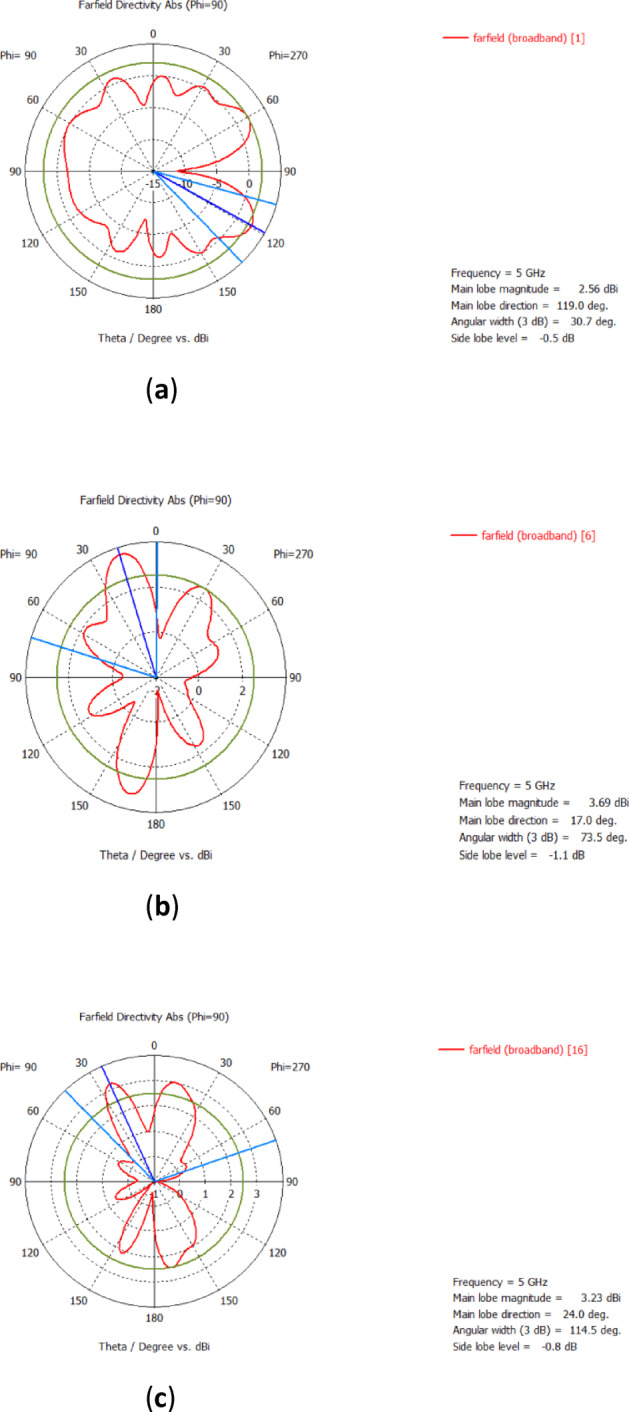
2D far-field radiation performance of some Antenna elements. (**a**) Ant 1, (**b**) Ant 6, (**c**) Ant 16.

Figure 7 shows the overall efficiency of the suggested ST MIMO array. Due to the symmetrical structure, only the simulated efficiencies of Antenna elements 1–4 are displayed. It is observed that each element’s efficiency is close to the others, and is approximately 90%. The 2D far-field radiation performance is shown in Fig. 8.

## Algorithm simulation results

In this section, computer simulations are used to demonstrate the performance metrics of the suggested SAC-based algorithm, if compared with the proposed BCD-based algorithm in^42^ for the same IRS-assisted CR system. Firstly, we display the SU attainable rate in the suggested IRS-aided downlink MIMO CR system using both algorithms under different varying parameters. Then, we indicate the performance convergence and the complexity of computations for both algorithms by comparing the required runtime under the same conditions. Also, the policy-learning efficiency of the SAC algorithm as a DRL algorithm is shown. The applied parameters of the SAC algorithm are shown in Table 1. Considering the case of perfect CSI, all SAC-based simulated figures include a BCD benchmark for performance comparison. Let the number of PU-RX, K = 2, be fixed for all figures.

We adopt the perfect CSI case in the simulations for simplicity, but our future work will be an extension to the current effort and will include a detailed comparison with simulated figures for both perfect and imperfect CSI cases. Actually, we should endorse the imperfect CSI case, but this is more complicated, and we have two main approaches. The stochastic approach models the CSI errors as Gaussian random variables, and the parametric constraints are modeled by the probability constraints. While the worst-case approach assumes that the CSI errors fall inside a specified uncertainty set, the optimization process is achieved under the worst-case channel conditions. We aim to employ the DRL algorithms to overcome such complexity in our future work.

**Table 1:** Parameters of SAC-based algorithm.

Parameter	Value
Q network learning rate	β = 0.0003
Policy network learning rate	β’ = 0.0001
Soft update learning rate	$\tau$ = 0.005
Temperature update	ψ = 0.05
Experience replay size	$\mathcal{P}$ = 200,000
Mini-batch size	${N}_{B}$ = 256
Target entropy	${H}_{0}= -\mathrm{dim}\left(action\right)$
Soft target adjustment factor	$\eta =30.$

**Fig. 9:**
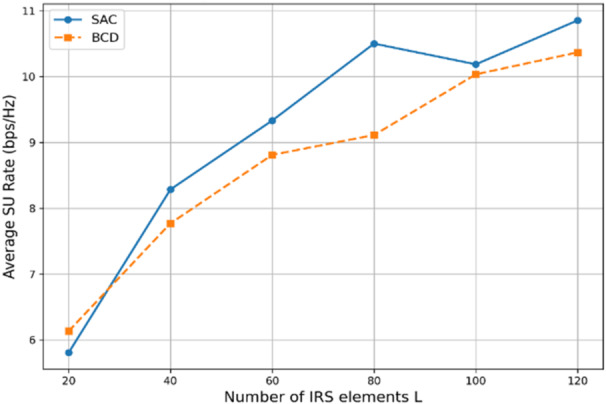
Achievable SU rate of both algorithms vs. L, when *P* = 6 dBm, *N* = 2, and Γ = 5 dB.

Figure 9 shows the achievable SU rate for both algorithms versus the number of reflecting elements in each IRS array. Using that, *N* = 2, Γ = 5 dB, *P* = 6 dBm, SAC episodes = 200, steps per episodes = 50 and BCD iterations = 20. It is obvious that the SU achievable rates increase with L for both algorithms. Furthermore, the curve of SAC rate almost outperforms that of BCD except at the beginning of the simulation, where the SAC algorithm has been in the exploration stage, learning for better and more stable actions. Thus, such DRL-based algorithms can accomplish higher SU rates if compared to traditional AO-based algorithms.

**Fig. 10:**
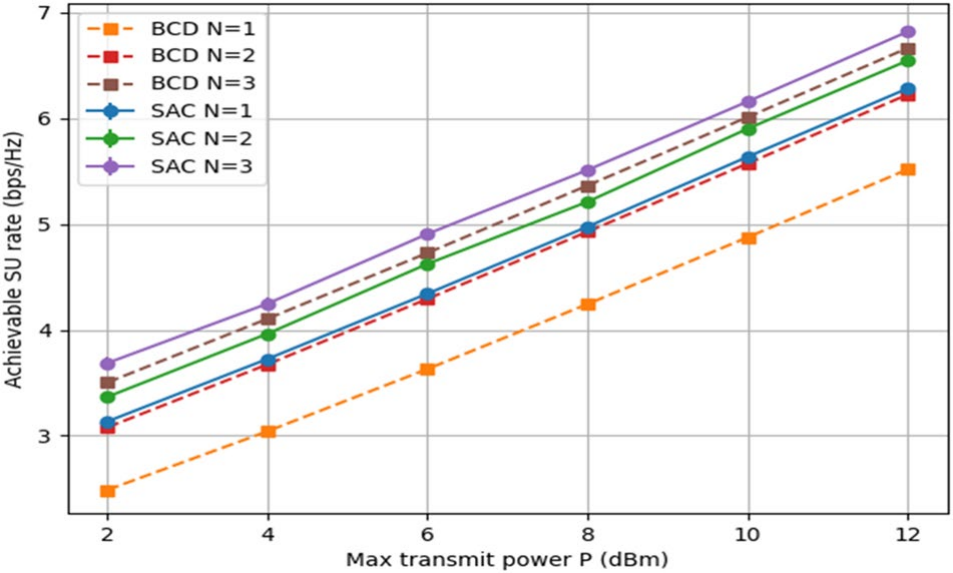
Achievable SU rate of both algorithms vs. P, when N ∈ {1, 2, 3}, Γ = 5 dB.

Figure 10 displays the SU attainable rate with respect to a varying transmitting power limit, P, when the IT constraint is fixed to Γ = 5 dB. Considering multiple IRS arrays, i.e., *N* = 3, while the number of RIS reflecting elements in each array is fixed, L = 10. Using that, SAC episodes = 200, steps per episode = 50, and BCD iterations = 20. As anticipated, the achievable rates of both algorithms increase with P, and, again, the SAC rate curves dominate.

**Fig. 11:**
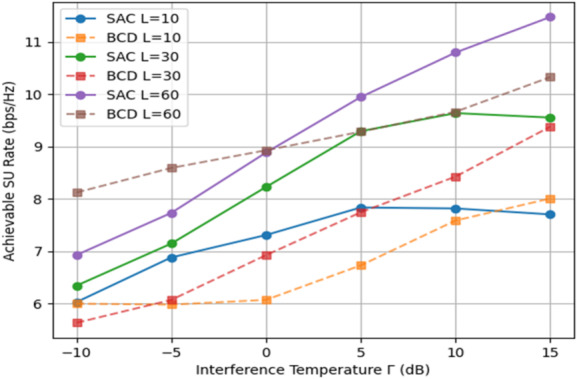
Achievable SU rate of both algorithms vs. Γ, when *N* = 2, *P* = 10 dBm and L ∈ {10, 30, 60}.

In Fig. 11, the SU achievable rate is calculated over the IT constraint Γ with a variable number of IRS elements when *P* = 10 dB. Using that, SAC episodes = 300, steps per episode = 50, and BCD iterations = 20. It can be observed that, when Γ rises, i.e., the interference limit is more flexible, the achievable SU rates of both algorithms increase. In addition, is obvious that the SU achievable rate of the IRS-assisted CR system significantly improves when L increases due to the fact that the IRS array is more effectively configured to steer the reflected wave in a desired direction when L is large enough.

**Fig. 12:**
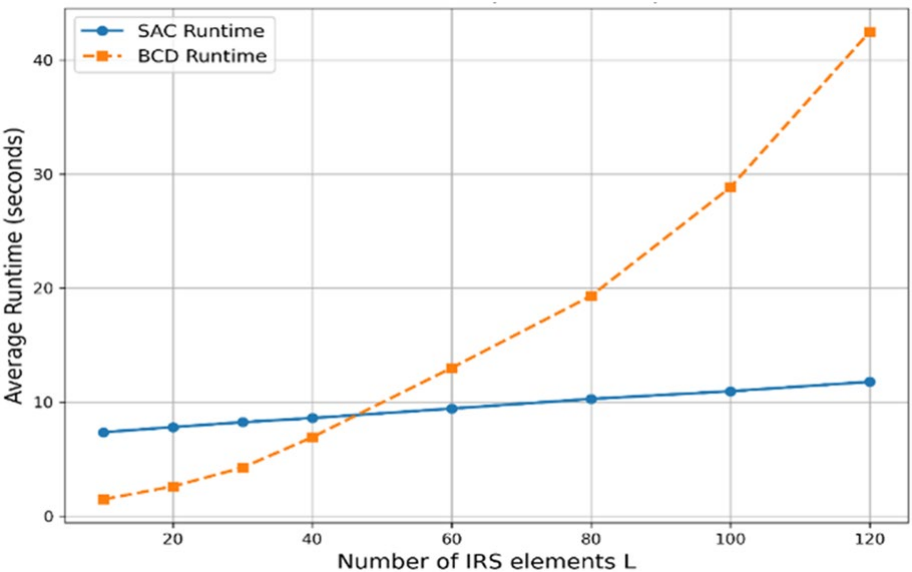
The average runtime of both algorithms vs. L, when *N* = 2, Γ = 5 dB.

The previous figures demonstrated that the SAC achievable SU rate obviously outperforms the BCD rates under the mentioned conditions. To indicate the computational complexity and the required execution time for both algorithms, Fig. 12 shows the average run time subjected to a varying number of IRS elements. Using that, SAC episodes = 500, steps per episode = 100, and BCD iterations = 30. It is observed that the run time of the SAC-based algorithm, which is an indication of the analysis complexity, is higher than BCD run time only if the utilized number of IRS elements is relatively small since SAC requires an exploration and learning stage before being stable and making a developed action with optimized phase shifts and corresponding transmit power while the BCD algorithm can iteratively optimize such a small number of elements rapidly. On the other hand, for larger L, SAC dominates since BCD requires more sequential time to iteratively optimize the IRS phase of all elements in each block before repeating this process for many iterations to achieve convergence.

**Fig. 13:**
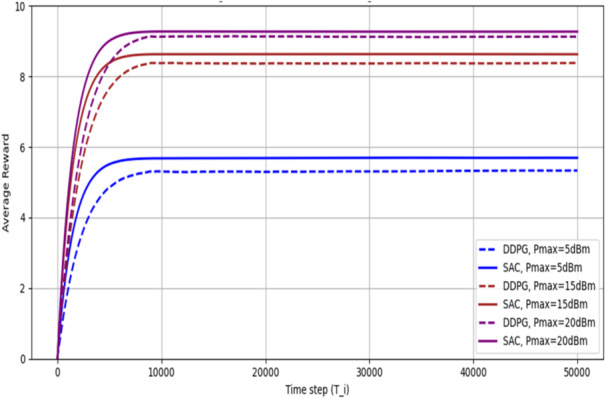
The average reward $\:\overline{R\left({T}_{i}\right)}$ of both SAC and DDPG-based algorithms for $\:{P}_{max}=5,\:15,\:20\:dBm.$.

After proving the superiority of DRL-based algorithms over the conventional AO-based algorithms, as shown in the previous figures, Fig. 13 concerns the efficiency and learning capability of the proposed SAC algorithm; it shows the average cumulative reward $\:\overline{R\left({T}_{i}\right)}$ with respect to the applied time steps at several values of $\:{P}_{max}$ in dBm. If compared with the DDPG-based algorithm in^32^, it is noticeable that the SAC algorithm usually achieves a higher average cumulative reward since its reward is less variant. This is due to the fact that the SAC agent develops a stochastic policy with a built-in action exploration capability. So, there is no need to manually add some noise as in the DDPG agent case. This advantage enables adjusting the action exploration process using an adaptive factor λ based on the environment parameters. In addition, if compared to the DDPG approach, the SAC algorithm’s average reward curves have comparatively sharper increasing slopes, indicating a higher learning efficiency. The considered average cumulative reward $\:\overline{R\left({T}_{i}\right)}$ is defined as:

$$\:{\overline{R\left({T}_{i}\right)}}=\frac{\sum\:_{t=1}^{{T}_{i}}r\left(t\right)}{{T}_{i}},\:{T}_{i}=\mathrm{1,2},3,\dots\:\dots\:\dots\:,T.$$

where *t* represents the current time step, *T* represents the maximum time step, and *r(t)* is the immediate reward value.

## Conclusion

This paper suggests an IRS-aided CR system to enhance the achievable SU rates without heavily interfering the network’s primary users. Then, the ST beamforming vector and the IRS reflection coefficients of each configured array have been jointly optimized subjected to specified IT limits at the PU-RXs and the total transmitting power constraint at the ST. Considering the perfect CSI case, we convert the maximization problem to a DRL problem, which can be solved by the SAC-based algorithm that guarantees a comparatively higher SU achievable rate with less complex analysis and high learning efficiency, if compared to the alternative conventional optimization methods, such as the BCD-based algorithm that was used as a benchmark in our comprehensive simulations. Besides these results, a 16-element ST base station MIMO array was proposed, practically manufactured, and tested. It has a 90% radiation efficiency, less than − 6 dB return losses, and below 0.02 ECC, which indicates an excellent performance. In our future research, it could be an interesting challenge to consider both perfect and imperfect CSI and compare the SAC-based algorithm with other pioneering DRL-based algorithms.

## Data Availability

The core of the empirical work consists of the simulation and analysis of data generated using Python within Visual Studio Code, as well as CST Studio simulations. This comprehensive dataset includes the source codes, numerical results, and output figures from computational models developed specifically to address the non-convex optimization problem which is formulated to enhance the achievable rate of the secondary user (SU) without exceeding both the transmitting power constraint of secondary base station and the interference temperature limit (IT) on the existing primary users (PU) in the proposed IRS-assisted cognitive radio network.For any data requests related to this study, please contact Rna Ghallab (ghallabrna@gmail.com).
